# Medical and Sanitary Legislation

**Published:** 1905-01

**Authors:** 


### TEXAS MEDICAL JOURNAL.

AUSTIN, TEXAS.

A MONTHLY JOURNAL OF MEDICINE AND SURGERY.

EDITED AND PUBLISHED BY
F. E. DANIEL, M. D.
Mrs. F. E. DANIEL, Business Manager.

Published Monthly at Austin, Texas. Subscription price $1.00 a year in advance.

Eastern Representative: John Guy Monihan, St. Paul Building, 220 Broadway New York City.

Official organ of the West Texas Medical Association, the Houston District Medical Association, the Austin District Medical Society, the Brazos Valley Medical Association, the Galveston County Medical Society, and several others.

### MEDICAL AND SANITARY LEGISLATION.

"Against stupidity even the Gods are powerless to contend."

An organization called "The Drugless Doctors' State Association of Texas" has sprung into existence for the avowed purpose of fighting the proposed amendments to the existing medical practice act. They have issued a declaration of war to the members of the Legislature, which is remarkable chiefly for illiteracy, mendacity, and brag. It maligns and villifies the State Medical Association of Texas, impugns its motives,—denounces its methods as a "dirty political trick"; repudiates vaccination, utterly scouts "infection," and laughs to scorn the use of antitoxin serums; says "layety," "outragious," "conspicious," "The Nazarine"; calls the Eclectics "the electrics," calls the Homœopaths "The Homopaths," etc. In short, it is a piece of stupid *blackguardism,* worthy only of the contempt of self-respecting persons. Of course, we can not stoop to a controversy with men who unblushingly make such misstatements and false accusations; but they must be corrected. We utterly repudiate the unworthy insinuations and deny the statements as to the motives and objects of the State Medical Association. Their president, a Dr. Teer of Tyler, in his manifesto, makes, however, one statement that I will bank on. It is true, but not in the sense intended. He says: "No drugless doctor would pass an examination before a board of medical doctors." That's no joke,—if they all spell as he does.

The following correspondence will be read with interest:

NAVASOTA, TEXAS, December 19, 1904.

*Dr. F. E. Daniel, President State Medical Association, Austin, Texas.*

DEAR SIR: I am in receipt of various communications from "The Drugless Doctors' Association" respecting proposed legislation in the Twenty-ninth Legislature.

I would be glad to have your side of the controversy.

Yours truly,

G. J. WINTER,

Representative Forty-fourth District.

STATE MEDICAL ASSOCIATION OF TEXAS.

OFFICE OF THE PRESIDENT.

AUSTIN, TEXAS, December 21, 1904.

*Hon. G. J. Winter, Representative in Twenty-ninth Legislature, Navasota, Texas.*

DEAR SIR: I have the honor to acknowledge the receipt of your letter of recent date, requesting me to give you my presentation of the cause of medical legislation, and stating that you are receiving numerous letters of protest from a class of doctors who call themselves the "Drugless Doctors' Association." In compliance with your request, I have the honor to submit herewith the facts in the case and the views of myself and those I represent, as to the necessity of a change in the medical practice law, and the enactment of sanitary legislation for the better protection of the public health.

Yours very truly,

F. E. DANIEL, M. D.

**A Brief for the People.**

The public health is the paramount interest in every State or nation. Upon it depend all other interests, and the prosperity, the progress,— even the perpetuation of the race. It is less safeguarded in Texas than any other interest, public or private, personal or property.

Sanitary science aims at the prevention of disease and injury, the protection of the public health by the removal of known and possible dangers to the individual or the community. The majority of diseases are preventable; and a death from any preventable disease is a crime chargeable against the State and a reproach to its civilization.

Quarantine is not the sum total of sanitation. The non-intercourse quarantine is a relic of barbarism. It is unnecessary. Sanitary science can prevent the spread of disease without destroying commerce and travel. But it must be enforced by the State; physicians, individually or collectively, can not do it unless armed with the authority and means.

The enlightened physician can and does instruct his clientelle how to avoid being sick, by the removal of the causes of disease,—the application of the principles of sanitary science and the rules of personal, household, business, and school hygiene. It is with them to heed him or not. So, the organized medical profession is logically the advisers of the government in the application of the principles of sanitation. State Medicine (synonyms, Public Hygiene, Preventive Medicine) is defined to be the application and enforcement by the State of the principles of sanitary science for the prevention of disease and injury, in the interest of the public. No intelligent legislation in the interest of the public safety can be enacted except upon the advice of the learned physicians of the State. It is with the lawmakers to accept or reject that advice.

The State Medical Association of Texas embraces 47.6 per cent (the best element) of the regular profession of the State. They have pointed out time and again that Texas loses annually thousands of its people from preventable diseases, and have asked, in the name of humanity, of public polity and social economy, that means of prevention be enforced. They have no private ends to subserve; indeed, they would, if they could, render their services as practitioners of medicine (healers of disease), unnecessary, by *preventing people from being sick!* The treating of disease is not all of "practicing medicine." Medical science embraces not only therapeutics, but sanitation as well. A learned physician has said: "The 'medicine' of the twentieth century will be sanitation and not 'physic.'" Nor is the giving of drugs the whole of medical practice. Of this, further on.

Consumption, for an illustration, is an infectious and communicable disease; but it is not contagious (catching). The communication of the disease from the sick to the well can be prevented by comparatively simple methods. But it requires authority and means to do it. For every death in the United States from yellow fever, there are 150 deaths from consumption. Those are the figures of the U. S. Census Reports. In 1900 there were in America 150,000

deaths from consumption,—a preventable disease; that is 411 a day, every day, all the time. In five years 750,000 deaths occur from consumption,—a mortality greater than that of the four years of the Civil War,—from all causes in both armies. This means a million and a half deaths from a preventable disease each decade! No efforts are made in Texas to prevent this appalling and unnecessary waste of life. And where one dies there are perhaps six suffering (and most likely will die), enfeebled, incapacitated, and communicating the disease to others. Thus the integrity of our social existence is threatened.

Inspection of foods, drinks, drugs, liquors, milk, water, etc., and the prevention of adulteration and pollution of the same, will largely prevent typhoid fever and diphtheria, dysentery and other intestinal diseases that carry off so many people. Sanitary policing of premises, public and private, schoolhouses and cars, especially, the great disseminators of infection, drainage, proper plumbing, ventilation, and disinfection at intervals, doubtless will prevent a score of other diseases. The adulteration and abuse of alcoholic liquor annually kills its quota, and the extensive and indiscriminate use of patent medicines containing alcohol, cocaine, opium, etc., is a menace to the public health far-reaching and fatal. *Blindness in a large per cent of cases is preventable by simple means.

All these matters should be in the hands of the most learned medical men in the State,—a Public Health Commission, or a State Board of Health, backed by the authority of law, and with ample means to enforce the necessary measures of prevention. The State Medical Association will ask the Thirtieth Legislature to enact such a law. The public health interest of Texas is too precious, too momentous and extensive, the population of the State is too great; our territory too vast to be supervised and the necessary sanitary laws administered and executed by any one man, however able, zealous and efficient; and I take pleasure in saying that Texas could not get a wiser, abler, more zealous and efficient man than the present head of the Public Health Department. He is handicapped, however, and this important service is crippled by lack of assistance and means.

But there are dangers to the public health other than disease,

*What do the care and education of the blind in Texas cost the State? A large number of children are made blind by infantile sore eyes (*ophthalmia neonatorium*). It is an infectious disease, caused by unclean midwifery—and requires prompt and intelligent antiseptic treatment. There should be no unclean midwifery and no infection in the vaginal tract and no ophthalmia; but so long as there are, blindness can only be prevented by the intelligent local use of antiseptics;—"drugs."

and they should be guarded against, and prevented. Thus, safeguards are thrown around every dangerous pursuit and occupation *except the most dangerous of all,* the privilege of dealing with life and death by the practice of medicine. The ignorant, so-called "doctor" is, indeed, a "terror,"—a large factor in the mortality rate, an unknown and unsuspected danger; hence its magnitude. True, the State requires of its own graduates and of the graduates of all reputable medical colleges, evidence of qualification, training and fitness before licensing them to practice medicine; but, strangely enough, it requires no such test and evidence from a large class of so-called doctors, upon the ground that they do not give drugs or medicines. Strange fallacy! As if the giving of drugs constituted the practice of medicine; and to not give drugs is to be *not* practicing medicine. Yet the courts of many States have held and ruled (notably the Supreme Court of Illinois, of Missouri, of Alabama), that those who do not give drugs or medicine, yet who undertake to treat disease and injury, are practicing medicine within the meaning of the law; and these "drugless" doctors claim that they are physicians; they offer for practice and take all cases, and take pay for their services. They claim that they are a school or schools of medicine, and that they have medical colleges, and that they are graduates of those colleges, and have medical diplomas, and that they are educated in all the branches of medicine, except chemistry, therapeutics, and practice. And yet, they are permitted, without license, to attend the sick and wounded. It is clearly the duty of the State to require of every person offering to treat disease by any method whatever, to give to the State satisfactory evidence of education, qualification and fitness for the exercise of so dangerous a privilege, before entrusting him or her with its exercise. The Medical Practice Act discriminates against the educated in favor of the uneducated. The Constitution says: "The State shall have the power to regulate the practice of medicine, but no preference shall be shown to any school of medicine." A decided "preference" is shown all the "drugless" class in that they are not required to be examined, as others are, for a license, or to have a license. The principles of sanitation apply to this, the uneducated, and, therefore, incompetent "doctor," as to all other dangers to the public health, and those of the profession who are seeking legislation to safeguard the public health would protect it from this as from all other dangers. Of course, they cry out "persecution," and impugn our motives, and falsify and misrepresent. "No thief ever felt the halter draw, and entertained a good opinion of the law," or words to that effect. The amendment to the Medical Practice Act, which the State Medical Association will present to the Twenty-ninth Legislature, strikes out the clause which exempts from the operation of the law those who do not give drugs or medicines, and thus brings them to a level with all other practitioners, except that it does not require that they shall stand examination upon therapeutics, practice, or chemistry. They,— the Osteopaths, Vitopaths, Magnetic Healers, *et id. om. gen.*, declare they are educated in all other branches of medicine. If they are, they should be examined upon those branches, and give to the State evidence of it, before being permitted to deal with life and death.

Their sin is often one of omission, but oftener, of commission. Take as an illustration of the former, a case related by Senator Savage, of Montague, of a healthy girl of eighteen who broke her thigh and was under the ministration of a Christian Science woman, who induced the family to refuse to have a surgeon. The girl died of blood poisoning. Illustrative of the "commission" sin, I cite a case well known about Austin, of a "drugless" one who, being called to a child with a fracture of the shoulder (neck of humerus), said it was "out of joint," and pulled on it, day after day, until the child's arm was in a horrible condition, and her life was saved by a surgeon, only by a scratch.

Christian Science people are practicing medicine and take pay for it. They do not claim to be educated in medicine, however, or to be graduates of medical schools. To require them to be examined in any of the branches of medicine would be tantamount to prohibiting it altogether, which is unnecessary, and would be eminently unjust. But they should be prohibited, under heavy penalty, from undertaking the treatment of any acute disease, or an injury, or attending women in labor, and should be required, under penalty for failure to do so, when called to any such case, to send for or have sent for, a licensed physician. In my judgment that is the solution of the Christian Science problem. Prayer for certain forms of ailment,—frequently imaginary,—does no harm and may, by suggestion and faith, do good and effect cures. There is nothing new or startling about faith-healing. It is true; the influence of suggestion on the body is well known, but not in infectious or contagious diseases, or any acute disease, or in surgery.

Prayer will not heal broken bones or prevent blood poisoning, nor cure diphtheria or smallpox.

An argument is offered that it is a personal right, guaranteed, to choose one's own medical attendant. No one disputes it. Every man has a right to employ whatever doctor he prefers. So he has a right to ride on a passenger train in charge of a blind engineer. But the State says a blind engineer shall not take out a passenger train. Every citizen has a right, unquestionably, to employ the doctor of his choice, but the *State should see that there are no ignorant and incompetent, and, therefore, deadly, doctors amongst those to be chosen from.*

I trust, sir, that I have presented the subject fairly and clearly, and that we may count on your intelligent and earnest co-operation in the great humanitarian work before us.

Very respectfully yours,

F. E. DANIEL, M. D,,

President State Medical Association and ex-officio Chairman Committee on Public Policy and Legislation; President International Congress on Tuberculosis, 1905-6; editor TEXAS MEDICAL JOURNAL, etc.

